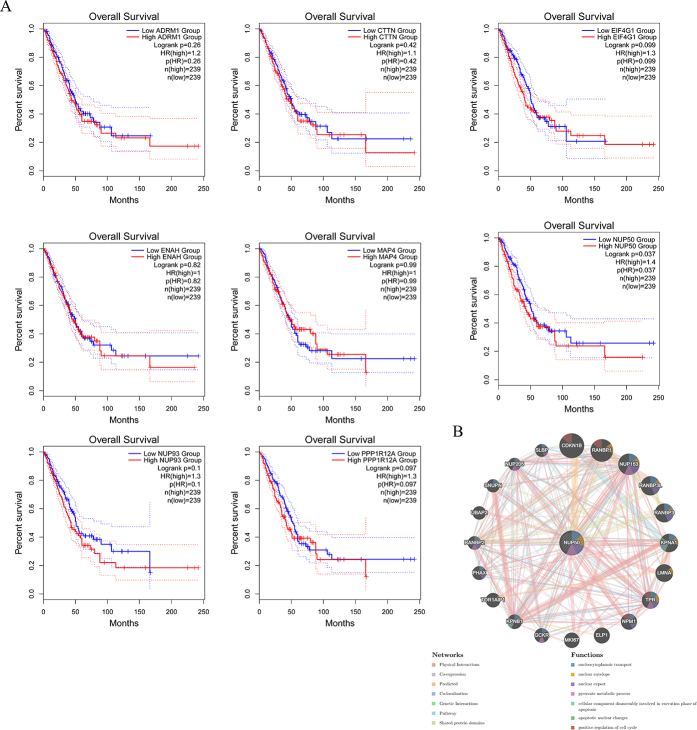# GALNT7-induced O-glycosylation of NUP50 activates fatty acid β-oxidation to promote lung adenocarcinoma metastasis

**DOI:** 10.1016/j.jbc.2026.113179

**Published:** 2026-05-22

**Authors:** Meiling Sheng, Beiwei Yu, Qunzhi Wang, Yuanchao Xiao, Xiaoming Wu

**Affiliations:** 1 Department of Respiratory and Critical Care Medicine, Jinhua Hospital Affiliated to Wenzhou Medical University, Jinhua, Zhejiang Province, China; 2 Department of Clinical Laboratory, Jinhua Hospital Affiliated to Wenzhou Medical University, Jinhua, Zhejiang Province, China; 3 Interventional Department, Jinhua Hospital Affiliated to Wenzhou Medical University, Jinhua, Zhejiang Province, China

**Keywords:** GALNT7, NUP50, O-glycosylation, metastasis, fatty acid β-oxidation

## Abstract

Lung adenocarcinoma (LUAD) is a common and highly metastatic subtype of lung cancer. Despite its high prevalence, the molecular pathways underlying its metastatic potential remain poorly elucidated. Here, we revealed increased GALNT7 expression and Tn antigen levels in LUAD tissues. Using a series of CCK-8, Transwell, and Western blot assays, we demonstrated that GALNT7 knockdown effectively inhibited LUAD cell proliferation, migration, and invasion, while also mitigating epithelial-mesenchymal transition and reducing metastasis-associated protein levels. In contrast, GALNT7 overexpression exacerbated these phenotypes. Mechanistically, GALNT7 colocalized with NUP50 at the nuclear envelope and enhanced the O-glycosylation of NUP50, thereby stabilizing the protein and activating fatty acid β-oxidation pathways, which are critical for LUAD cell metastasis. Knockdown of GALNT7 disrupted this pathway, markedly inhibiting metastasis. *In vivo*, using nude mouse xenograft and lung metastasis models, we confirmed that GALNT7 overexpression can synergize with NUP50-WT to significantly promote tumor growth and metastasis, while also enhancing the levels of NUP50 and VVA. In contrast, supplementation with NUP50-MUT effectively blocked the tumor-promoting effects of GALNT7 overexpression. In summary, this study systematically dissects the role of GALNT7 in LUAD metastasis, revealing key mechanisms that could inform the development of new therapeutic strategies and potential drug targets.

Lung adenocarcinoma (LUAD) is the most prevalent form of non-small cell lung cancer (NSCLC), representing approximately 40% of all lung cancer diagnoses (1). Despite the evolution of treatment paradigms from traditional chemotherapy to personalized therapies that leverage the genetic profiles of cancer cells, which has improved patient outcome (2), the overall survival (OS) of patients with LUAD remains unsatisfactory. Notably, the 5-year survival rate for advanced LUAD remains low, primarily due to metastasis and recurrence after surgery (3, 4). These clinical challenges highlight the necessity for deeper insights into the mechanisms driving LUAD metastasis, which could open new avenues for therapeutic development.

Glycosylation is a fundamental post-translational modification that fine-tunes protein function. Mucin-type O-glycosylation, which targets serine and threonine residues, is a key feature of many membrane and secreted proteins, and this modification is essential for regulating protein stability, cell signaling pathways, and various biological processes (5). Alterations in mucin-type O-glycosylation are increasingly recognized as key contributors to various cancers, including lung cancer (6, 7). Truncated O-glycosylation (Tn antigen or O-GalNAcylation) is often overexpressed in cancer cells and is associated with metastasis and poor survival. For example, Li *et al.* (8) demonstrated that Tn-mediated aberrant O-glycosylation drives breast cancer metastasis by disrupting CASC4 expression and function. These findings underscore the potential of targeting glycosylation in cancer therapy (9). GALNTs are a family of enzymes that initiate mucin-type O-glycosylation (10). Their dysregulation can impact the malignant behaviors of cancer cells. Hu *et al.* (11) reported that GALNT2 is highly expressed in NSCLC and promotes tumor progression by modifying the O-glycosylation of ITGA5.

To date, researchers have identified 20 members of the GALNT family in the human genome, spanning from GALNT1 to GALNT20. Among these enzymes, our study focuses on GALNT7, an enzyme that catalyzes the attachment of N-acetyl galactosamine (GalNAc) to serine or threonine residues in proteins (12). microRNAs can downregulate GALNT7 expression, thereby inhibiting the progression of lung cancer and the proliferation and invasion of cervical cancer (13, 14). Additionally, GALNT7 has been reported to modify the O-glycosylation of cell surface and secreted proteins, promoting prostate cancer growth (7). The above evidence implicates GALNT7-mediated O-glycosylation in the metastasis and progression of multiple cancers, pointing to its potential as a key oncogenic factor. Nonetheless, the specific role and mechanisms of GALNT7 in LUAD are not fully understood.

Using bioinformatics and cellular experiments, we analyzed the expression of GALNT7 and O-glycosylation levels in LUAD and examined the role of GALNT7 in LUAD cell migration, invasion, epithelial–mesenchymal transition (EMT), and metastasis. We identified NUP50 as a substrate of GALNT7 in LUAD and showed that GALNT7-mediated O-glycosylation of NUP50 promotes tumor metastasis through the fatty acid β-oxidation (FAO) signaling pathway. This study not only sheds light on the metastatic mechanisms of LUAD but also underscores the potential for translating this knowledge into clinical applications to develop new LUAD therapies.

## Results

### Elevated GALNT7 and Tn antigen levels in LUAD

**Figure 1:**
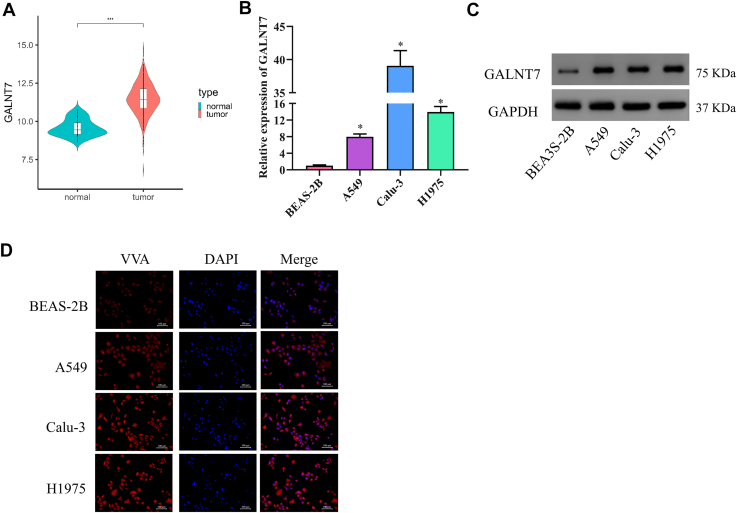
**Expression of GALNT7 and Tn antigen levels in LUAD.***A*, higher GALNT7 expression in tumor tissues compared to normal tissues revealed by TCGA data. *B* and *C*, GALNT7 mRNA and protein levels assessed in LUAD cells and normal BEAS-2B cells using RT-qPCR and WB. *D*, Immunofluorescence detection of Tn antigen levels in LUAD cells and their normal counterparts (scale bar = 100 μm). ∗ indicates *p* < 0.05.

TCGA-LUAD database analysis identified that LUAD tissues had higher GALNT7 expression than normal tissues (Fig. 1*A*). RT-qPCR and WB analyses showed that GALNT7 mRNA and protein levels were evidently higher in LUAD cell lines A549, Calu-3, and H1975 compared to BEAS-2B cells (Fig. 1, *B* and *C*). Immunofluorescence analysis of VVA, which specifically targets the Tn antigen often associated with cancer progression (15), revealed pronounced upregulation of Tn antigen levels in LUAD cells (Fig. 1*D*). These findings suggest that LUAD is marked by elevated GALNT7 expression and increased levels of abnormal mucin-type O-glycans.

### GALNT7 promotes LUAD cell proliferation, migration, and invasion

**Figure 2:**
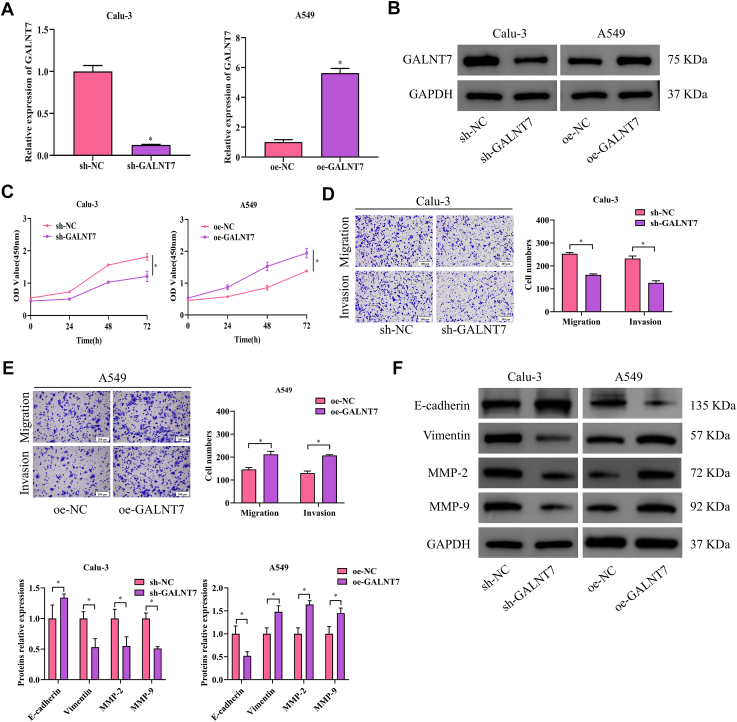
**The role of GALNT7 in LUAD cell metastasis.***A* and *B*, GALNT7 knockdown (sh-NC/sh-GALNT7) and overexpression (oe-NC/oe-GALNT7) confirmed by RT-qPCR (*A*) and WB (*B*). *C*, cell proliferation measured using the CCK-8 assay. *D* and *E*, cell migration and invasion evaluated using Transwell assays (scale bar = 200 μm). *F*, the expression of EMT and metastasis-related proteins revealed by WB analysis. ∗ indicates *p* < 0.05.

**Figure S1:**
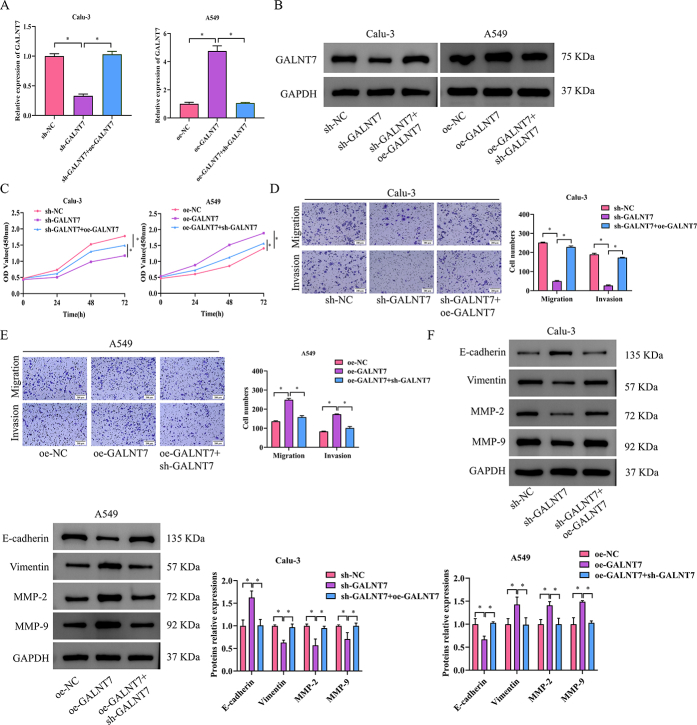


To elucidate the biological function of GALNT7 in LUAD, we constructed cell models with GALNT7 knockdown, overexpression, and rescue experiments. GALNT7 knockdown and rescue models (sh-NC, sh-GALNT7, sh-GALNT7+oe-GALNT7) were established in Calu-3 cells, while overexpression and rescue models (oe-NC, oe-GALNT7, oe-GALNT7+sh-GALNT7) were established in A549 cells. RT-qPCR and WB confirmed that GALNT7 expression was effectively regulated in all groups (Figs. 2, *A* and *B* and S1, *A* and *B*). CCK-8 assays showed that knocking down GALNT7 significantly inhibited the proliferation of Calu-3 cells, while overexpressing GALNT7 promoted the proliferation of A549 cells (Fig. 2*C*); these effects could be reversed by subsequent overexpression or knockdown (Fig. S1*C*). Transwell assays further demonstrated that GALNT7 knockdown impaired the migration and invasion of Calu-3 cells, a phenotype that could be rescued by re-expression; conversely, GALNT7 overexpression enhanced the motility of A549 cells, an effect that could be suppressed by subsequent knockdown (Figs. 2, *D* and *E*, and S1, *D* and *E*). EMT is a key driver of tumor progression, invasion, and metastasis in cancer (16). Matrix metalloproteinases (MMPs), such as MMP-2 and MMP-9, degrade type IV collagen to facilitate cancer cell metastasis (17). We also investigated whether GALNT7 affects the expression of EMT and metastasis-related proteins. WB analysis of EMT and metastasis-related proteins revealed that in Calu-3 cells, GALNT7 knockdown led to the upregulation of E-cadherin and downregulation of Vimentin, MMP-2, and MMP-9, changes that were reversed upon rescue expression. In A549 cells, GALNT7 overexpression induced the opposite trends, which could be restored by subsequent knockdown (Figs. 2*F* and S1*F*). Thus, we inferred that GALNT7 plays a crucial role in LUAD cell proliferation, migration, and invasion.

### GALNT7 regulates NUP50 stability *via* O-glycosylation

**Figure 3:**
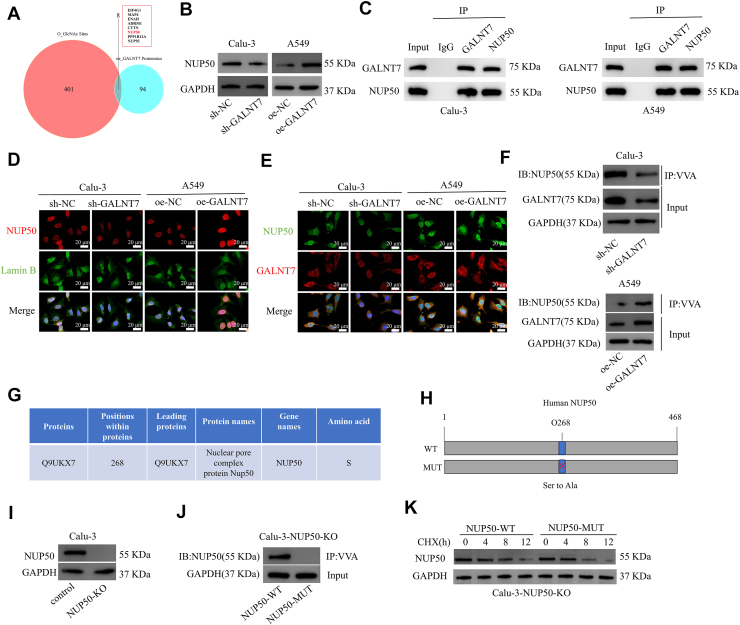
**GALNT7 modifies NUP50 *via* O-glycosylation and regulates its protein levels.***A*, Venny analysis showing the overlap between high-confidence O-GlcNAc sites and proteomic data from GALNT7-overexpressing tumor cells. *B*, WB confirming altered NUP50 expression in LUAD cells with GALNT7 knockdown or overexpression. *C*, Co-IP assay for detecting the interaction between GALNT7 and NUP50; *D*, immunofluorescence analysis of the colocalization between NUP50 and Lamin B after GALNT7 knockdown and overexpression. *E*, immunofluorescence analysis of the colocalization between GALNT7 and NUP50 after GALNT7 knockdown and overexpression. *F*, VVA precipitation assays showing that NUP50 binding is affected by GALNT7 knockdown or overexpression, as detected by immunoblot; *G*: O-glycosylation sites on NUP50 mapped using the O-GlcNAc dataset. *H*, mutants of NUP50 with altered O-glycosylation sites; I: NUP50-KO Calu-3 cells created using CRISPR/Cas9. *J*, VVA precipitation assays showing differences in NUP50 binding between NUP50-WT and NUP50-MUT cells. *K*, altered NUP50 protein stability revealed by CHX chase assays combined with WB analysis. ∗ indicates *p* < 0.05.

Building on the work of Qin *et al.* (18), who developed a new method for O-GlcNAc biology research, we acquired a dataset of high-confidence O-GlcNAc sites. By intersecting this dataset with the proteomics data from GALNT7-overexpressing tumor cells (7), we identified eight potential target genes (Fig. 3*A*). To identify key targets, we performed survival analysis on the TCGA-LUAD cohort using the GEPIA2 online tool. Only high expression of NUP50 was significantly associated with shorter OS in patients, while the remaining seven candidate genes had no significant prognostic value (Fig. S2*A*). Functionally, as a single gene cannot undergo conventional enrichment analysis, we used the GeneMANIA platform to construct a co-expression interaction network for NUP50. This network visualization clearly indicated that the core functions of NUP50 are significantly associated with biological processes such as nucleocytoplasmic transport (Fig. S2*B*). Therefore, we identified NUP50 as the most promising downstream target of GALNT7 for subsequent mechanistic studies. WB results revealed that GALNT7 knockdown in Calu-3 cells suppressed NUP50 expression, whereas GALNT7 overexpression in A549 cells enhanced NUP50 expression (Fig. 3*B*). These results suggest that GALNT7 can regulate NUP50 expression in LUAD cells. To further elucidate the specific mechanism by which GALNT7 regulates NUP50, we first performed co-IP assays and confirmed a robust endogenous interaction between GALNT7 and NUP50 in two LUAD cell lines (Fig. 3*C*). Subsequent immunofluorescence assays revealed that GALNT7 knockdown reduced the distribution of NUP50 at the nuclear envelope, whereas GALNT7 overexpression promoted the enrichment of NUP50 in the perinuclear and nuclear envelope regions. Although GALNT7 is a glycosyltransferase residing in the Golgi lumen, immunofluorescence analysis clearly detected its distribution in the perinuclear region with obvious spatial overlap with NUP50. Moreover, the expression level of GALNT7 was positively correlated with the colocalization intensity of NUP50 (Fig. 3, *D* and *E*). We sought to confirm if GALNT7 modifies the O-glycosylation of NUP50 by conducting lectin pull-down experiments with biotinylated VVA and streptavidin-agarose in LUAD cells where GALNT7 was either knocked down or overexpressed. The pulled-down glycoproteins were subjected to immunoblot using an anti-GALNT7 antibody. The data indicated that GALNT7 knockdown decreased the expression of NUP50 in the glycoprotein fraction captured by VVA (Fig. 3*F*). On the other hand, GALNT7 overexpression resulted in elevated O-glycosylation levels of NUP50 (Fig. 3*F*). These findings highlight the essential role of GALNT7 in the O-glycosylation of NUP50 in LUAD cells.

To determine the glycosylation sites of NUP50 in LUAD cells, we analyzed a high-confidence O-GlcNAc dataset and identified Ser268 as a potential O-glycosylation site (Fig. 3*G*). Using this information (Fig. 3*H*), we engineered a mutant NUP50 by substituting serine at position 268 with alanine. We then used sgRNA to create an NUP50 knockout (NUP50-KO) in Calu-3 cells (Fig. 3*I*). The wild-type NUP50 (NUP50-WT) and the mutant NUP50 (NUP50-MUT) constructs were stably introduced into this cell model. VVA precipitation assays showed that NUP50 glycosylation was completely lost in NUP50-MUT cells (Fig. 3*J*). To shed light on the potential mechanism, we probed into the influence of O-glycosylation on NUP50 protein stability. The data indicated that, when protein synthesis was blocked by CHX, NUP50 degradation in LUAD cells was accelerated by the NUP50-MUT mutation (Fig. 3*K*). In summary, these findings point to the stabilizing effect of GALNT7-mediated O-glycosylation on NUP50 in LUAD cells.

### NUP50 O-glycosylation fuels LUAD metastasis *via* FAO

**Figure 4:**
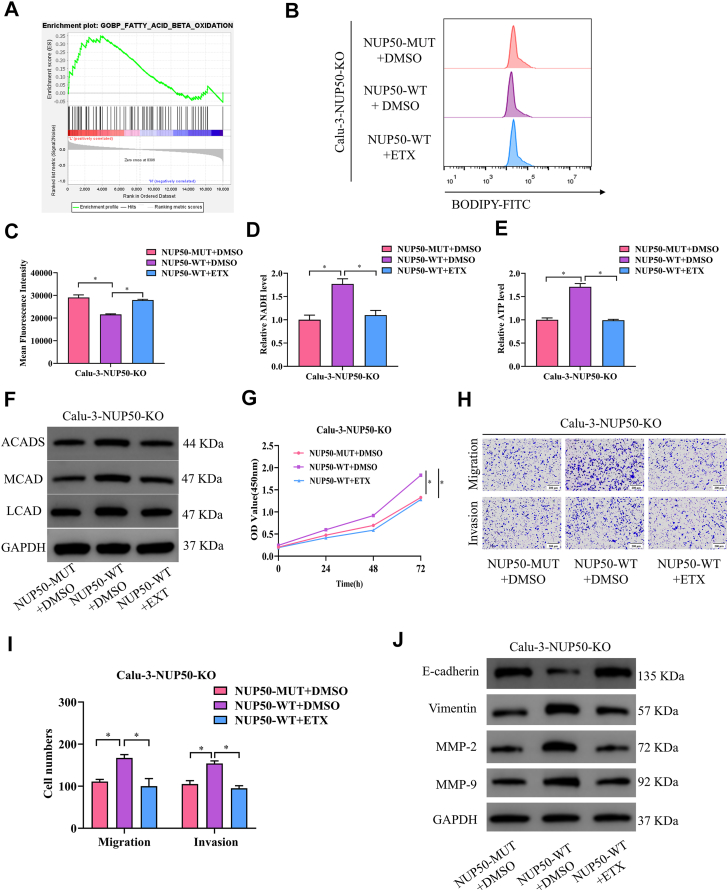
**O-Glycosylation of NUP50 plays a crucial role in LUAD cell metastasis.***A*, Pathway enrichment analysis of NUP50 using GSEA. *B* and *C*, Neutral lipid droplet content measured by flow cytometry in NUP50-KO Calu-3 cells with wild-type or mutant NUP50 treated with DMSO or ETX. *D* and *E*, NAD+/NADH and ATP levels were measured using assay kits to assess metabolic activity. *F*, expression of ACADS, MCAD, and LCAD analyzed by WB. *G–I* proliferation (CCK-8 assay), migration, and invasion (Transwell assays) were evaluated in NUP50-KO Calu-3 cells (H: scale bar = 200 μm). *J*, EMT and metastasis-related proteins detected by WB. ∗ indicates *p* < 0.05.

Previous research has demonstrated that NUP50 can drive cancer progression (19). To explore the role of O-glycosylation in NUP50-mediated LUAD progression, we performed GSEA, revealing that NUP50 was predominantly enriched in the FAO pathway (Fig. 4*A*), which suggests that FAO could dictate the oncogenic function of NUP50. Building on these findings, we established three experimental groups using NUP50-KO Calu-3 cells: NUP50-MUT+DMSO, NUP50-WT+DMSO, and NUP50-WT+ETX. Flow cytometry showed that glycosylated NUP50 dramatically reduced neutral lipid content compared to the NUP50-MUT+DMSO group. Nonetheless, this reduction was rescued by the FAO inhibitor ETX (Fig. 4, *B* and *C*). NADH and ATP levels were markedly elevated in NUP50-WT+DMSO cells relative to NUP50-MUT+DMSO cells, according to the data yielded using assay kits. ETX treatment, however, greatly diminished these levels in cells with glycosylated NUP50 (Fig. 4, *D* and *E*). WB analysis of FAO-related genes (ACADS, MCAD, and LCAD) also showed that ETX partially reversed the upregulation of these genes by glycosylated NUP50 (Fig. 4*F*). These results suggest that O-glycosylation of NUP50 could enhance FAO. We further assessed the impact of NUP50 O-glycosylation on LUAD cell proliferation, migration, and invasion using CCK-8 and Transwell assays. The results showed that NUP50-WT cells exhibited remarkably enhanced capabilities in these aspects compared to NUP50-KO Calu-3 cells with a glycosylation site mutation. However, ETX treatment partially mitigated these effects (Fig. 4, *G*–*I*). WB analysis revealed that NUP50-WT+DMSO cells had elevated levels of Vimentin, MMP-2, and MMP-9, while E-cadherin was downregulated. In contrast, cells treated with ETX exhibited restored levels of EMT and metastasis-related proteins comparable to those in NUP50-MUT+DMSO cells (Fig. 4*J*). Collectively, these findings suggest that NUP50 O-glycosylation could promote LUAD cell metastasis *in vitro* by activating FAO.

### GALNT7 knockdown rescues LUAD cell metastasis mediated by NUP50 glycosylation

**Figure 5:**
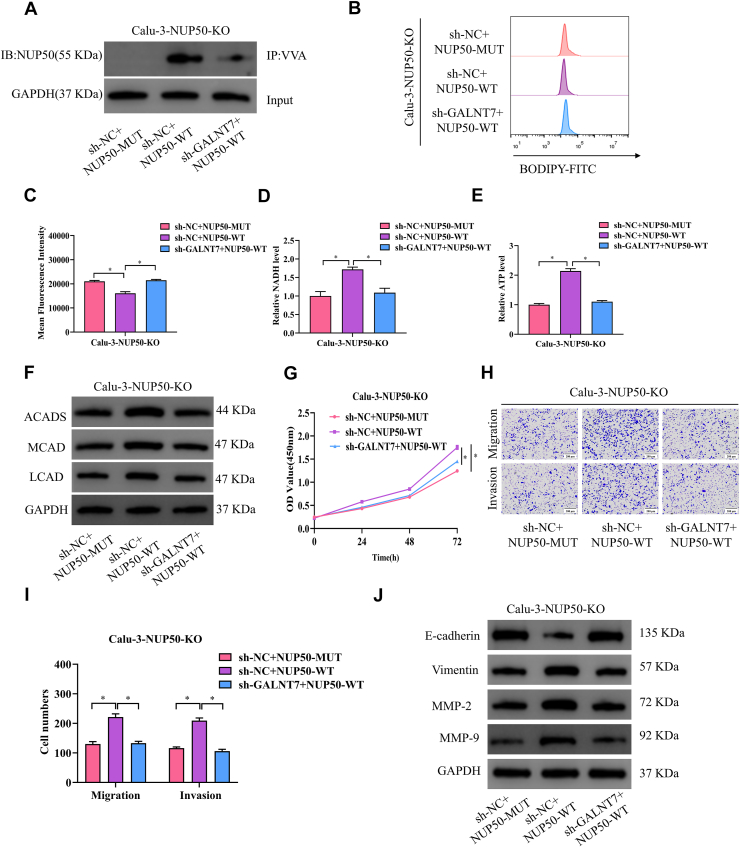
**GALNT7 knockdown rescues LUAD cell metastasis mediated by NUP50 glycosylation.***A*, VVA precipitation assays evaluating the impact of GALNT7 on NUP50 glycosylation in NUP50-KO Calu-3 cells: sh-NC+NUP50-MUT, sh-NC+NUP50-WT, or sh-GALNT7+NUP50-WT. *B* and *C*, neutral lipid droplet content quantified by flow cytometry. *D* and *E*, NADH (*D*) and ATP (*E*) levels in LUAD cells measured using NAD+/NADH and ATP content assay kits. *F*, ACADS, MCAD, and LCAD expressions analyzed by WB. *G–I*, proliferation (CCK-8), migration, and invasion (Transwell assays) assessed in NUP50-KO Calu-3 cells. *J*, EMT and metastasis-related proteins detected by WB. ∗ indicates *p* < 0.05.

Given that GALNT7 acts as an upstream glycosyltransferase inducing abnormal O-glycosylation of NUP50 in LUAD cells, we sought to determine its role in the metastatic function mediated by NUP50 glycosylation. To this end, we stably transfected NUP50-KO Calu-3 cells, which either harbored a glycosylation site mutation or expressed wild-type NUP50, with sh-GALNT7 or a negative control. VVA precipitation assays showed that GALNT7 knockdown led to a marked reduction in NUP50 glycosylation levels in NUP50-WT cells (Fig. 5*A*). Flow cytometry further revealed that while NUP50-WT transfection decreased neutral lipid droplet content, sh-GALNT7 transfection resulted in a considerable increase (Fig. 5, *B* and *C*). Assay results showed that glycosylated NUP50 cells had higher NADH and ATP levels compared to non-glycosylated NUP50 cells. However, GALNT7 knockdown effectively eliminated this effect, restoring NADH and ATP levels in LUAD cells (Fig. 5, *D* and *E*). Similar trends were observed in the expression of FAO-related proteins (ACADS, MCAD, and LCAD) (Fig. 5*F*). These findings suggest that GALNT7 knockdown could inhibit FAO in LUAD cells by preventing the O-glycosylation of wild-type NUP50. CCK-8 and Transwell assays revealed that knocking down GALNT7 effectively counteracted the enhancement of LUAD cell proliferation, migration, and invasion by NUP50-WT (Fig. 5, *G*–*I*). WB analysis showed that compared to sh-NC+NUP50-MUT cells, sh-NC+NUP50-WT cells exhibited notably higher levels of Vimentin, MMP-2, and MMP-9, while E-cadherin was markedly reduced. However, these effects were abolished when GALNT7 was knocked down (Fig. 5*J*). Overall, these findings indicate that GALNT7-mediated O-glycosylation of NUP50 promotes LUAD cell metastasis by activating FAO.

### *In vivo* validation of GALNT7-driven LUAD growth and metastasis *via* NUP50 O-glycosylation

**Figure 6:**
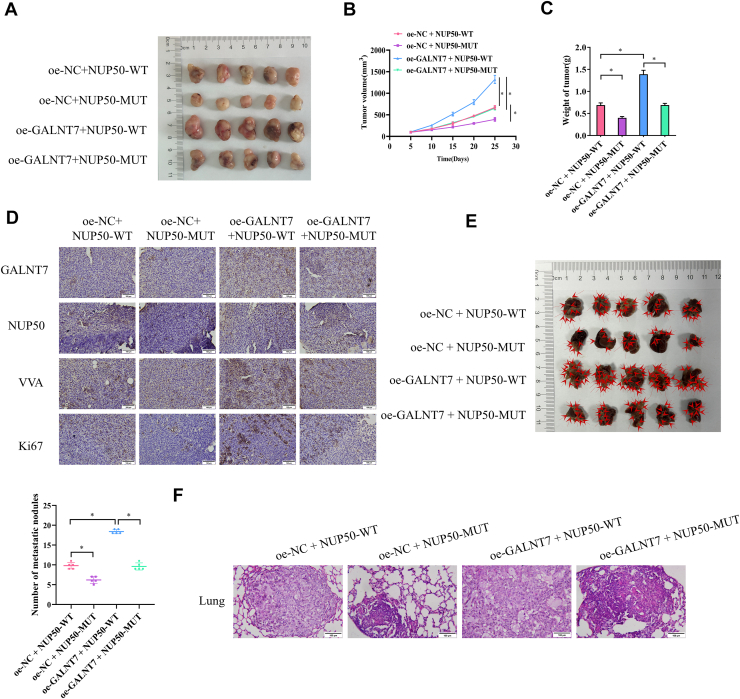
***In vivo* validation of the mechanism by which GALNT7 promotes LUAD metastasis.***A*, representative subcutaneous xenograft tumors (n = 5). *B* and *C*, Tumor volume and weight measurements. *D*, IHC detection of GALNT7, NUP50, VVA, and Ki67 in tumor tissues (scale bar = 100 μm); E: A549 cells (oe-NC+NUP50-WT/oe-NC+NUP50-MUT/oe-GALNT7+NUP50-WT/oe-GALNT7+NUP50-MUT) were injected into nude mice tail veins (n = 5), with lung images taken 4 weeks later; F: H&E-stained lung sections from nude mice (scale bar = 100 μm). ∗ indicates *p* < 0.05.

To validate the function of the GALNT7-NUP50 pathway *in vivo*, we constructed four stable A549 cell lines: oe-NC+NUP50-WT, oe-NC+NUP50-MUT, oe-GALNT7+NUP50-WT, and oe-GALNT7+NUP50-MUT, and performed subcutaneous tumor formation and tail vein lung metastasis model experiments. Subcutaneous tumor formation results showed that the oe-GALNT7+NUP50-WT group exhibited the most rapid tumor growth, with final tumor volume and weight significantly higher than those of the other three groups. In contrast, tumor growth in the oe-GALNT7+NUP50-MUT group was significantly inhibited, with volume and weight showing no statistical difference compared to the oe-NC+NUP50-WT control group (Fig. 6, *A*–*C*). Furthermore, the oe-GALNT7+NUP50-WT group displayed the highest accumulation of GALNT7 and NUP50 proteins, VVA (Tn antigen) signal, and Ki67 positivity rate in tumor tissues. However, although the oe-GALNT7+NUP50-MUT group showed high GALNT7 expression, its NUP50 accumulation, VVA signal, and proliferative activity were significantly reduced (Fig. 6*D*).

We further confirmed *via* the lung metastasis model that the oe-GALNT7+NUP50-WT group had the highest number of metastatic nodules on the lung surface (Fig. 6*E*), and H&E staining revealed the highest degree of infiltration by metastatic lung nodules in this group (Fig. 6*F*). In contrast, the metastatic ability of the oe-GALNT7+NUP50-MUT group was substantially weakened, with the number of metastatic nodules comparable to that of the oe-NC+NUP50-WT group. These *in vivo* experiments indicate that the pro-tumor growth and metastasis function of GALNT7 is strictly dependent on its O-glycosylation modification of NUP50.

## Discussion

Metastasis remains the primary cause of mortality in LUAD patients, underscoring the pressing need to further investigate the regulatory networks that govern LUAD metastasis (20). Our study provides compelling evidence from both *in vivo* and *in vitro* models that the glycosyltransferase GALNT7 plays a pivotal role in LUAD metastasis. We demonstrate that GALNT7 is highly expressed in LUAD and drives LUAD metastasis by catalyzing the O-glycosylation of NUP50. This modification could stabilize NUP50 by inhibiting proteasomal degradation. Importantly, knocking down GALNT7 could reduce glycosylated NUP50 levels and suppress LUAD metastasis *via* fatty acid oxidation pathways. Our findings highlight the GALNT7-NUP50 axis as a critical driver of LUAD progression.

GALNT7 is known to be upregulated in various malignancies, including lung cancer, luminal breast cancer, and prostate cancer, where its overexpression is associated with cancer progression (13, 21, 22). In papillary thyroid carcinoma (PTC), miR-30b-5p targets GALNT7 to obstruct the EGFR/PI3K/AKT pathway, resulting in reduced PTC proliferation, migration, and invasion (23). However, the precise role and underlying mechanisms of GALNT7 in LUAD remain elusive. Echoing prior findings, our study revealed that GALNT7 was markedly overexpressed in LUAD tissues and cells, as confirmed through bioinformatics analysis and cellular assays, which suggests that GALNT7 might serve as a pivotal oncogene in tumor progression. Through a series of *in vitro* experiments, we also found that elevated GALNT7 expression could drive LUAD cell proliferation, migration, invasion, EMT, and the upregulation of metastasis-related proteins. Evidence suggests that elevated GALNT7 expression in prostate cancer leads to increased levels of Tn antigen (7). Tn antigen, a well-known tumor-associated glycan, has emerged as a therapeutic target across multiple cancers (24). Numerous studies have reported that Tn antigen expression is upregulated in various cancers and is associated with tumor metastasis (8, 25). Our findings, supported by the use of VVA lectin, reveal elevated Tn antigen levels in LUAD cells, consistent with earlier studies. The above data suggest that GALNT7 and its associated glycans could be promising candidates for LUAD treatment. Future work should focus on in-depth mechanistic characterization to establish GALNT7 as a viable target for LUAD treatment and prognosis.

To investigate the mechanisms underlying LUAD metastasis mediated by GALNT7, we pinpointed NUP50 as a downstream target by intersecting differentially upregulated proteins in GALNT7-overexpressing cancer cells with a high-confidence dataset of O-GlcNAc sites. Nucleoporins, part of the nuclear pore complex (NPC), exhibit cell type-specific functions (26) and impact cancer development through their role in nucleocytoplasmic transport (27, 28). NUP50, a nucleoporin, is essential for nucleocytoplasmic transport, a critical function of the NPC (29). NUP50 has surfaced as a pivotal protein in cancer dynamics, particularly metastasis. Research has repeatedly shown that NUP50 is upregulated in cancers such as gastric and esophageal cancer, and its surge is tightly linked to how long patients survive and whether the cancer spreads far and wide (30, 31). Despite the cancer relevance of nucleoporins, the mechanisms driving high NUP50 expression in cancer cells remain unclear. In this study, we identified that GALNT7 induces O-glycosylation of NUP50 in LUAD cells. As a canonical mucin-type O-glycosyltransferase predominantly localized within the Golgi lumen, GALNT7 typically targets transmembrane or secreted proteins in the secretory pathway as its classical substrates (32). Combined results from co-IP and immunofluorescence assays confirmed the colocalization of GALNT7 and NUP50 in the perinuclear and nuclear envelope regions, providing crucial spatial evidence for the modulatory effect of GALNT7 on NUP50. CHX chase analysis demonstrated that this modification stabilizes NUP50 by blocking proteasomal degradation. These results verified the hypothesis that O-glycosylation of NUP50 is a key contributor to LUAD metastasis.

Next, through GSEA analysis, we found that NUP50 was enriched in the FAO signaling pathway. FAO is a multistep process that degrades fatty acids to generate energy, producing key molecules such as NADH and ATP (33). This pathway is often dysregulated in various cancers, correlating with mechanisms of cancer cell proliferation, stemness, and metastasis (34). PGC1α promotes TGFβ1-induced EMT and metastasis in nasopharyngeal carcinoma cells through FAO (35). In recent years, research has revealed that enzymes involved in FAO, including ACADS, LCAD, and MCAD, contribute to the progression of colorectal and breast cance (36, 37). We developed LUAD cell lines expressing glycosylated and non-glycosylated NUP50 based on reported O-glycosylation sites. Rescue experiments using ETX, a clinically approved FAO inhibitor that effectively suppresses tumor growth and metastasis (38, 39), showed that glycosylated NUP50 enhanced LUAD cell metastasis, an effect partially mitigated by ETX. These findings illuminate the role of NUP50 O-glycosylation in promoting LUAD cell metastasis *via* FAO.

O-glycosylation, mediated by glycosyltransferases, is necessary for the functional activity of various specific substrates (40, 41). GALNT7, a key player in this process, is essential for glycoprotein processing and stability, thereby fueling tumor growth (7). Our findings show that GALNT7 interacts with NUP50, but whether GALNT7 can O-glycosylate NUP50 to influence LUAD cell metastasis is yet to be determined. In the current study, silencing GALNT7 in glycosylated NUP50-overexpressing cells abolished the enhanced metastatic ability and FAO in LUAD cells. Xenograft models in nude mice showed that GALNT7 knockdown clearly inhibited tumor growth and the expression of NUP50 and VVA proteins. In the metastasis model, GALNT7 knockdown also appreciably reduced the number of pulmonary metastatic nodules. These results elucidate the critical role of GALNT7-mediated aberrant NUP50 O-glycosylation in promoting LUAD metastasis.

This study has several limitations. It relies on public databases and lacks validation with our own clinical data. Future work should include prospective analysis to explore GALNT7 expression levels in LUAD patients. Additionally, the mechanism by which glycosylated NUP50 regulates FAO remains unclear. Further research is needed to clarify this mechanism, including whether NUP50 interacts with ACADS, LCAD, or MCAD.

Our study identifies a novel mechanism by which GALNT7 upregulation contributes to LUAD metastasis *via* aberrant O-glycosylation of NUP50, leading to enhanced FAO. These findings have significant translational potential, suggesting that targeting GALNT7 or glycosylation pathways could offer a new therapeutic approach to inhibit LUAD metastasis and improve patient outcomes.

## Experimental procedures

### Bioinformatics

mRNA expression data for LUAD and normal tissues (normal: 70; tumor: 859) were downloaded from the TCGA database. Using *edgeR*, we conducted differential expression analysis (|logFC| > 1, FDR < 0.05) to identify differentially expressed mRNAs (DEmRNAs). We intersected these DEmRNAs with high-confidence O-GlcNAc sites from a published dataset (18) and proteins upregulated in tumor cells from a proteomic analysis (42). Next, to assess the clinical significance of the candidate genes, survival analysis was performed on the TCGA-LUAD cohort using the GEPIA2 online tool to compare the correlation between the expression levels of each candidate gene and the OS of patients. Additionally, to elucidate the biological functions of the candidate genes, a gene interaction network was constructed using the GeneMANIA platform to visualize their co-expression relationships and functional annotations. We then divided patients with LUAD into two groups based on the median expression of NUP50 and performed gene set enrichment analysis (GSEA) to explore gene function enrichment.

### Cell culture and transfection

Human normal epithelial cells (BEAS-2B) and three LUAD cell lines (A549, Calu-3, and H1975) were obtained from SUNNCELL. All cell lines were authenticated by the supplier using short tandem repeat profiling prior to purchase and are regularly tested to maintain their authenticity. BEAS-2B, A549, and Calu-3 cells were cultured in DMEM medium (Gibco) containing 10% fetal bovine serum (FBS; Gibco) and 1% penicillin-streptomycin (Gibco). H1975 cells were cultured in RPMI-1640 medium (Gibco) with the same supplements. All cells were maintained at 37 °C in a humidified incubator with 5% CO_2_.

The sh-GALNT7 (sense: GATCCCGCCCATTTATGTTGGGTCTTCTCGAGAAGACCCAACATAAATGGGCGTTTTTG; antisense: AATTCAAAAACGCCCATTTATGTTGGGTCTTCTCGAGAAGACCCAACATAAATGGGCGG) and sh-NC lentiviruses were generated by GeneChem. When Calu-3 and A549 cells reached approximately 80% confluence, they were transfected with the lentiviruses. After 24 h, the medium was replaced with fresh complete medium to support stable integration. Puromycin (MCE) was then used to select stably transfected cells for subsequent assays. GALNT7 overexpression plasmids (oe-GALNT7) and negative controls (oe-NC) were obtained from GeneChem. Transfections were carried out using Lipofectamine 3000 (Invitrogen), and cells were incubated for 24 to 48 h prior to further experimental procedures. To investigate metabolic effects, Etomoxir (ETX, MCE) was dissolved in DMSO and applied to LUAD cell cultures at 30 μM for 24 h (43).

### Gene knockout *via* CRISPR-Cas9

To create a NUP50-deficient cell model, the human NUP50 CRISPR/Cas9 knockout plasmid (sc-406550) was purchased from Santa Cruz Biotechnology (USA). The plasmid was transfected into LUAD cells using UltraCruze Transfection Reagent (sc-395739, Santa Cruz Biotechnology, USA) as recommended by the manufacturer. The CRISPR/Cas9 control double-nicking plasmid (sc-418922, Santa Cruz Biotechnology) served as a negative control, and stable knockout cells were selected using puromycin (sc-1080711, Santa Cruz Biotechnology).

### qPCR

Total RNA was isolated from cells using TRIzol (Invitrogen) and converted into cDNA *via* reverse transcription using the Hifair II first Strand cDNA Synthesis Kit (YESEN). qPCR was conducted on an Applied Biosystems 7500 Fast instrument (Thermo Fisher) with the uGreener Flex qPCR 2× Mix (U&G BIO). The expression levels of GALNT7 were normalized to GAPDH using the 2^−ΔΔCt^ method. Primer sequences are listed in Table 1.

**Table 1:** Primer set for qPCR

Gene	Primer sequence (5′→3′)
GALNT7	F: AGATGAGGCTGAAGATTGGG
	R: GGAAGACCAGAGGACCACTA
GAPDH	F: CGGGAAGCTTGTCATCAAT
	R: TCTCCATGGTGGTGAAGA

### Western blot (WB)

Log-phase LUAD cells were lysed in RIPA buffer containing protease inhibitors (Beyotime) on ice for 30 min. The lysates were then centrifuged at 12,000 rpm for 15 min, and the supernatants were collected. Protein concentrations were determined using a BCA protein quantification kit (Solarbio). For SDS-PAGE analysis, 20 μg of protein was loaded per lane and separated, followed by wet transfer to PVDF membranes (MilliporeSigma). Membranes were blocked with 5% skim milk for 1 h at room temperature, then incubated with primary antibodies overnight at 4 °C. Antibodies used included GALNT7 (ABclonal, A20259), NUP50 (ABclonal, A9441, China), E-cadherin (ABclonal, A20798), Vimentin (Proteintech, 60330-1-Ig), MMP-2 (ABclonal, A19080), MMP-9 (ABclonal, A25299), ACADS (ABclonal, A20926), MCAD (ABclonal, A4567), LCAD (ABclonal, A4884), Vicia Villosa Lectin (VVA) (Vector Laboratories, CAT# B-1235–2), and GAPDH (HUABIO, ET1601–4). After the initial incubation, the membrane was washed three times with TBST for 5 min each. The membrane was then incubated with HRP-conjugated secondary antibodies (goat anti-rabbit IgG, Beyotime, A0208; or goat anti-mouse IgG, Beyotime, A0216) at room temperature for 1 h. Following another three 5-min washes with TBST, the membrane was treated with an ultra-sensitive ECL chemiluminescent substrate (Beyotime, P001S) for signal detection. The signal was captured with a ChemiScope 6200 imager (Clinx).

### Cell proliferation assessment

LUAD cells were seeded into a 96-well plate at 3 × 10^3^ cells per well and cultured for 0, 24, 48, and 72 h. Cell viability was then measured using the CCK-8 assay (APE x BIO). Specifically, 10 μl of CCK-8 solution was added to each well and incubated at 37 °C for 2 h. Absorbance was measured at 450 nm using a Multiskan FC microplate reader (Thermo Fisher), and the proliferation rate was calculated based on these readings.

### Transwell assays

For invasion assays, Transwell inserts (Corning) were coated with Matrigel matrix (Corning, USA) to mimic the extracellular matrix barrier, while uncoated inserts were used for migration assays. LUAD cells (2 × 10^4^) were suspended in 200 μl of serum-free medium and added to the upper chamber, with 600 μl of complete medium containing 10% FBS in the lower chamber. After incubation at 37 °C for 24 h (migration) or 48 h (invasion), the inserts were fixed with 4% paraformaldehyde for 15 min and stained with 0.1% crystal violet for 30 min. The migrated or invaded cells were visualized and counted under a Leica microscope.

### Quantification of neutral lipid droplets *via* flow cytometry

Neutral lipid droplets were detected using BODIPY 493/503 staining (Beyotime). Cells were harvested by trypsinization, washed once with PBS, and stained with 0.5 ml of staining solution for 10 to 15 min at room temperature in the dark. Following a PBS wash, the cells were analyzed by flow cytometry (Agilent), and the data were processed using FlowJo V10 software.

### Measurement of cellular NADH and ATP

NADH and ATP levels were measured using the NAD+/NADH Assay Kit (Solarbio, BC0315, China) and the ATP Content Assay Kit (Solarbio, BC0300, China). Cells were seeded into a 96-well plate at 1 × 10^4^ cells per well and cultured overnight. The next day, cells were lysed with extraction buffer and sonicated for 1 min on ice (200W, 2-s pulses). After centrifugation at 10,000g for 10 min at 4 °C, the supernatant was collected and analyzed using a Multiskan FC microplate reader (Thermo Fisher) at 450 nm and 340 nm.

### Co-immunoprecipitation (CO-IP)

Calu-3 and A549 cells in the logarithmic growth phase were collected and washed twice with pre-cooled PBS. Cells were lysed in pre-cooled RIPA lysis buffer supplemented with 1 mM PMSF and protease inhibitor cocktail on ice for 30 min. After centrifugation at 12,000 rpm for 15 min at 4 °C, the supernatant was harvested, and the protein concentration was quantified using the BCA method. A portion of the cell lysate was retained as the Input sample, mixed with 4× SDS-PAGE loading buffer, denatured at 95 °C for 5 min, and stored at −20 °C for subsequent use. A total of 500 μg of protein lysate was incubated with 2 μg of rabbit anti-GALNT7 antibody (NBP2-97010F, Novus Biologicals), rabbit anti-NUP50 antibody (A301-783A, Thermo Fisher), or rabbit IgG isotype control (MA5-42729, Thermo Fisher) respectively, with gentle rotation overnight at 4 °C. Subsequently, 30 μl of Protein A/G agarose beads were added and incubated for 4 h under gentle rotation at 4 °C. The beads were collected by centrifugation at 3000 rpm for 1 min, and the supernatant was discarded. After washing the beads with pre-cooled RIPA lysis buffer, 40 μl of 1× SDS-PAGE loading buffer was added, followed by denaturation at 95 °C for 5 min. The supernatant was collected after centrifugation for subsequent WB detection.

### Immunofluorescence

Cells were seeded at 5 × 10^4^ per well in a 6-well plate containing glass slides and cultured overnight. On the second day, the medium was discarded, and cells were fixed with 75% ethanol for 30 min, followed by three 5-min washes with PBS. Cells were then permeabilized with 0.1% Triton X-100 (Sangon) for 10 min. After blocking with 5% BSA (Sangon, China) for 1 h at room temperature, cells were incubated overnight at 4 °C with VVA (Vector Laboratories, 1:200, CAT# B-1235-2). The next day, cells were incubated with Alexa Fluor 555-Streptavidin (maokangbio) for 1 h at room temperature. Cells were counterstained with DAPI (Solarbio, C0065) and imaged using a fluorescence microscope (KEYENCE).

For colocalization detection, cells from different groups were fixed, permeabilized, and blocked. Subsequently, the samples were incubated overnight at 4 °C with primary antibodies, including mouse anti-GALNT7 (ab254971, Abcam), rabbit anti-NUP50 (ab137092, Abcam), mouse anti-NUP50 (H00010762-B01P, Thermo Fisher, USA), and rabbit anti-Lamin B (ab133741, Abcam). After three washes with PBS, the corresponding fluorescent secondary antibody Alexa Fluor 488 goat anti-rabbit (ab150077, Abcam) was added and incubated for 1 h at room temperature in the dark. Finally, cells were counterstained with DAPI (C0065, Solarbio), and images were captured using a fluorescence microscopy system (KEYENCE, Japan).

### Lectin sedimentation assay

To examine the variations in O-glycans, cell lysis was achieved using RIPA lysis buffer (Beyotime). Immunoprecipitation was performed on 1 mg of the total cell lysate with VVA linked to 40 μl Streptavidin-Agarose (Sigma). The immune complexes were ultimately analyzed through WB.

### Cycloheximide (CHX) chase assay

NUP50-WT and NUP50-MUT cells were treated with 20 μM CHX (MCE, USA) to assess the half-life of the NUP50 protein. Proteins were collected at 0, 4, 8, and 12 h post-CHX treatment before WB analysis.

### Immunohistochemistry (IHC)

Paraffin-embedded LUAD tissue sections were prepared and dewaxed using xylene, followed by rehydration. Antigen retrieval was performed using EDTA buffer. After washing with PBS, the sections were treated with an endogenous peroxidase inhibitor for 10 min at room temperature. The sections were then incubated overnight at 4 °C with primary antibodies against GALNT7 (ABclonal) and NUP50 (ABclonal). On the second day, they were incubated with HRP-conjugated goat anti-rabbit IgG (Beyotime) for 30 min at 37 °C, followed by diaminobenzidine (DAB) staining. After counterstaining with hematoxylin, the sections were dehydrated through graded alcohols, cleared with xylene, and examined under a microscope. For VVA detection in LUAD tissue, sections were blocked with Carbo-Free Blocking Solution (Neobioscience) for 30 min at room temperature to prevent nonspecific binding. The sections were then incubated overnight at 4 °C with VVA (Vector Laboratories) and subsequently with streptavidin-HRP (Sigma) for 1 h at room temperature.

### Hematoxylin and eosin (H&E) staining

Lung tissues from nude mice were fixed in 4% paraformaldehyde for 24 h, then dehydrated through a series of graded alcohols. The tissues were embedded in paraffin and sectioned at 5 μm. The sections were stained with hematoxylin and eosin (H&E) and imaged using a microscope (Mshot, China).

### Subcutaneous tumor model in nude mice

Twenty 4- to 5-week-old BALB/c nude mice were obtained from Hangzhou ZiYuan Laboratory Animal Technology Co., Ltd. For the subcutaneous tumor model, 100 μl of PBS containing 2 × 10^6^ A549 cells (oe-NC+NUP50-WT/oe-NC+NUP50-MUT/oe-GALNT7+NUP50-WT/oe-GALNT7+NUP50-MUT) was injected into the right flank of each mouse, with 5 mice per group. The body weight and tumor size were measured every 5 days, and all mice were euthanized under deep anesthesia after 25 days. The final tumor volume and weight were recorded using the formula: Volume = (length × width^2^)/2. For the pulmonary metastasis experiment, 100 μl of PBS containing 2 × 10^6^ A549 cells (oe-NC+NUP50-WT/oe-NC+NUP50-MUT/oe-GALNT7+NUP50-WT/oe-GALNT7+NUP50-MUT) was injected *via* the tail vein, with 5 mice per group. The mice were euthanized 4 weeks later, and the lungs were examined to count the metastatic nodules. The animal experiments were approved by the Ethics Committee of Zhejiang Luoxi Medical Technology Co, Ltd(No. LX4824121201).

### Statistical analysis

Statistical analysis was performed using GraphPad Prism 8. Data are presented as mean ± standard deviation (SD). Group comparisons were made using Student’s *t* test or one-way ANOVA. Significance was defined as *p* < 0.05.

## Ethics approval and consent to participate

This study was approved by the Animal Ethics Committee of Zhejiang Luoxi Medical Technology Co, Ltd, Hangzhou, China (No. LX4824121201), approval date 2024-12-12.

## Consent to participate statement

Patient consent was not required in accordance with local or national guidelines.

## Data availability

The data and materials in the current study are available from the corresponding author on reasonable request.

## Supporting information

This article contains supporting information (44,45).

## Conflict of interest

The authors declare that they have no conflicts of interest with the contents of this article.

## Figures and Tables

**Figure S2:**