# Supplementary material for: GALNT7-induced O-glycosylation of NUP50 activates fatty acid β-oxidation to promote lung adenocarcinoma metastasis
Source: J Biol Chem. 2026 May 22;302(7):113179. doi: 10.1016/j.jbc.2026.113179 (PMC13279022; doi:10.1016/j.jbc.2026.113179)
Supplement: Supplementary figures legend [file mmc1.docx]

**Figure S1: GALNT7 regulates the proliferation, migration, invasion, and EMT process of LUAD cells.**A-B: RT-qPCR (A) and WB (B) analysis of GALNT7 mRNA and protein expression levels in Calu-3 and A549 cells; C: Cell proliferation measured using the CCK-8 assay; D-E: Cell migration and invasion evaluated using Transwell assays (scale bar = 200 μm); F: WB analysis revealing the expression of EMT and metastasis-related proteins. * indicates *P* < 0.05.

**Figure S2: Identification of NUP50 as a key downstream target of GALNT7.**

A: Kaplan-Meier overall survival analysis of eight candidate genes in the TCGA-LUAD cohort; B: NUP50 functional interaction network diagram constructed using GeneMANIA.
